# Polypoidal choroidal vasculopathy—characteristics and response to treatment with bevacizumab in caucasian patients

**DOI:** 10.1186/s40942-022-00432-x

**Published:** 2022-11-22

**Authors:** Fidaa El Zhalka, Elad Moisseiev, Alexander Rubowitz

**Affiliations:** 1 Department of Ophthalmology, Meir Medical Center, 59, Tshernichovsky St, 4428164 Kfar Saba, Israel; 2 Sackler School of Medicine, Tel Aviv University, Tel Aviv, Israel

## Abstract

**Purpose:**

To investigate the features and treatment response in Caucasian patients with polypoidal choroidal vasculopathy (PCV), initially treated with bevacizumab.

**Methods:**

45 eyes of 43 treatment-naïve patients with PCV were included in this retrospective study, all uniformly initially treated with three bevacizumab injections monthly. OCT characteristics and clinical parameters were recorded and analyzed at presentation, after the initial 3 bevacizumab injections and at the final follow up period.

**Results:**

Following 3 monthly bevacizumab injections visual acuity significantly improved with a mean gain of one line of vision. Central macular thickness (CMT) significantly improved from a mean of 402.1 ± 130.8 μm at presentation to 322.0 ± 96.8 μm (p < 0.01). Subretinal fluid, intraretinal fluid and submacular hemorrhage significantly improved. 53% were later switched to aflibercept and showed better response in the central macular thickness in comparison to those in which bevacizumab injections were continued. No correlation was found between the presence of pachyvessels or increased choroidal thickness and the improvement in VA or CMT.

**Conclusion:**

Fixed first-line treatment with intravitreal bevacizumab monotherapy in non-Asian PCV patients achieves satisfactory visual and anatomical outcomes**.**

## Introduction

Polypoidal choroidal vasculopathy (PCV) is considered to be a subtype of neovascular AMD, however, some controversy exists about whether it should be classified as a distinct disease entity due to the difference in demographic characteristics, clinical and imaging features, disease course and treatment from other subtypes of NAMD [1]. In PCV type 1 neovascularization is associated with an abnormal branching network of vessels (branching vascular network [BVN]), with aneurysmal dilations referred to as *polyps* [2]. Accurate estimates of PCV prevalence are limited because of difficulties in diagnosing PCV from clinical examination and fundus photographs. In Asian patients with neovascular AMD, the proportion of PCV has been estimated to be between 20 and 60% [3]. In contrast, in Caucasians the proportion of PCV has been reported to be 8–30% of cases [4–7].

ICGA is considered the most reliable means for diagnosing PCV, however, it is not performed routinely in many parts of the world, particularly in areas where the disease is uncommon. Fluorescein angiography (FA), an imaging technique more commonly used in the diagnosis of exudative AMD, does reveal the presence of subretinal leakage and neovascularization, but lacks the ability to clearly visualize the terminal aneurysmal lesions required to make a PCV diagnosis in many cases. De Salvo et al [8] reported that based on a combination of 3 of the following 4 OCT-based signs—multiple pigment epithelial detachments (PEDs), sharp PED peak, PED notch, and rounded sub-RPE hyporeflective area—they were able to diagnose PCV based on OCT with a sensitivity of 94.6% and specificity of 92.9%. In another study, a combination of 2 of the following 3 signs—PED, double-layer sign, and thumb-like protrusion—was reported to detect PCV with a sensitivity of 89.4% and specificity of 85.3%. These studies suggest that SD OCT signs can be very helpful as a screening tool in areas where ICGA is not performed routinely [9].

Enhanced depth imaging optical coherence tomography (EDI-OCT) using spectral-domain optical coherence tomography (SD-OCT) is a non-invasive technique for visualizing choroidal structures. EDI-OCT shows that the subfoveal choroidal thickness (SFCT) is substantially different among the different subtypes of AMD, and that SFCT is greater in eyes with PCV than in eyes with typical AMD [10–12]. Thus, the pathogenesis of PCV has been linked to choroidal congestion leading to an increase in choroidal thickness [13]. The presence of a thick and hyperpermeable choroid in patients with PCV also suggests a link between PCV and the pachychoroid disease spectrum [14]. However, it has been shown that choroidal thickness is variable in eyes with PCV, with no statistically significant difference in the clinical and imaging features and treatment outcomes between eyes with thin, medium and thick SFCT patients with PCV according to some study [15] while others showed that in PCV eyes, thick choroids were associated with poor anatomical outcomes whereas eyes with thin choroids showed the greatest extent of improvement in anatomical but not visual outcomes [16]. Other studies indicate the opposite, that PCV eyes with thin choroid tend to be associated with older age and show less visual improvement after the initial three consecutive anti-VEGF treatments [17].

Results of the EVEREST-II and PLANET studies showed that anti-VEGF monotherapy (ranibizumab and aflibercept) as well as combination therapy with PDT gives excellent functional visual outcomes at 1 year, and is thus an acceptable initial treatment option in patients with symptomatic PCV [18, 19]. However, other studies have reported a higher incidence of anti-VEGF resistance in eyes with PCV [20, 21], suggesting that alternative treatments to anti-VEGF monotherapy may be preferred in these cases [22].

The purpose of this study was to investigate the characteristics and response to treatment in Caucasian patients with PCV, and specifically to initial treatment with bevacizumab. Additionally, rates of treatment resistance were evaluated, as well as the correlation between choroidal thickness and treatment response.

## Methods

A retrospective review of the electronic medical records of the ophthalmology department at the Meir Medical Center was conducted for patients diagnosed with PCV between 2005 and 2020. The study was approved by the Institutional Review Board of Meir Medical Center.

Patients treated before for PCV were excluded. Patients undergone previously ocular surgery or trauma, except for uncomplicated cataract extraction, or patients with prior vision limiting ocular conditions were excluded. All patients were 18 years or older, the diagnosis of PCV was made be a retina specialist, based on clinical examination and OCT, FA and/or ICGA.

Due to the limitations and regulations of all medical insurance carriers in Israel, which mandate first-line treatment with Bevacizumab treatment, all patients were initially treated uniformly with 3 monthly injections of bevacizumab. Patients who did not complete the first 3 months of treatment, missed or delayed injections, or who had an overall follow-up of less than 4 months after presentation were excluded. After follow-up examination after these injections, bevacizumab treatment was continued or changed at the discretion of the treating retinal specialist. Patients with incomplete records were also excluded.

Recorded parameters included demographic data, general medical history, ocular history, and lens status. Visual acuity (VA) was recorded at presentation, 1 month after 3 monthly injections of bevacizumab and at the final follow up visit. At these timepoints, OCT images were also analyzed for choroidal thickness, central macular thickness (CMT), presence of pachyvessels, presence of hemorrhages, intraretinal fluid (IRF) and subretinal fluid (SRF). Additionally, the types and numbers of different anti-VEGF injections were recorded, as well as any additional treatment, surgery or complication that occurred throughout the follow up period.

All visual acuity (VA) values were converted to the logMAR scale for statistical analysis. According to the results of Holladay and the University of Freiburg studies, counting fingers was set at 0.014/1.85, hand movements at 0.005/2.3, light perception at 0.0025/2.6 and blindness at 0.00125/2.9 (decimal/logMAR). To analyze categorical parameters chi-square tests were used, to continuous parameters between groups T tests were used. To analyze changes in VA and CMT over time Paired t tests were used. Correlations between the permanent variables were analyzed using Pearson’s correlation coefficient. Data was analyzed using SPSS for windows version 21. A p value of 0.05 was used to show the statistically significant difference between groups.

## Results

45 eyes of 43 patients who were treated in our clinic between January 1st, 2005 and December 31st, 2020 were included in the study. These included 21 (48.8%) men and 22 (51.2%) women, with a mean age at presentation of 75 ± 8.4 years (range 56–93 years). The most common systemic comorbidities were hypertension (55.8%) and diabetes mellitus (30%). The mean follow up period was 10.3 ± 6.5 months.

### Analysis of clinical characteristics at presentation

Twenty-five (55.5%) eyes were phakic at presentation, and the remaining 20 (44.5%) eyes were pseudophakic. The diagnosis of PCV was made by a retina specialist based on OCT characteristics alone in 23 (51.1%) eyes, based on OCT and FA in 9 (20%) eyes, based on OCT and ICG in 3 (6.6%) eyes, and based on all three imaging modalities at presentation in 13 (22.3%) eyes.

In OCT, the mean central macular thickness was 402.1 ± 130.8 µm, mean choroidal thickness was 228 ± 76.9 µm, and pachyvessels were present in 35 (77.3%) eyes. Subretinal fluid was present in 39 (86.4%) eyes, intraretinal fluid was present in 13 (27.3%) eyes, and submacular hemorrhage was present in 5 (11.4%) eyes.

### Analysis of response to initial bevacizumab treatment

All patients were treated with a fixed initial course of 3-month bevacizumab injections, and re-examined 4 weeks following the third injection. Visual acuity significantly improved from a mean of 0.59 ± 0.5 logMAR at presentation to 0.49 ± 0.5 (p = 0.009), indicating a mean gain of one line of vision after this initial treatment. Central macular thickness significantly improved from a mean of 402.1 ± 130.8 μm at presentation to 322.0 ± 96.8 μm (p = 0.00).

Subretinal fluid presence was improved from 86.4% to 47.7% (p = 0.022). Intraretinal fluid presence improved from 27.3% to 13.5% (p < 0.001) and submacular hemorrhage also improved after the three injections from 11.4%to 9.10% (p = 0.01) These results are presented in Table 1. The presence of pachyvessels did not changes during this time.

**Table 1:** visual acuity and OCT signs at presentation and during the follow up

	At Presentation	After three bevacizumab injections	Final
VA (LogMar)	0.59 ± 0.5	0.49 ± 0.5 (p = 0.009)	0.59 ± 0.6 (p = 0.698)
CMT (μm)	402.1 ± 130.8	322.0 ± 96.8 (p = 0.00)	305.2 ± 108.5 (p = 0.00)
Choroidal thickness (μm)	228 ± 76.9	216.5 ± 66.0 (p = 0.041)	223.1 ± 86.1 (p = 0.457)
SRF	86.4%	47.70% (p = 0.022)	25.50% (p = 0.312)
IRF	27.30%	13.60% (p = 0.00)	27.90% (p = 0.079)
submacular Hemorrhage	11.4%	9.10% (p = 0.00)	0% (p = 0.00)
Pachyvessels	77.30%	77.30%	77.30%

**Fig. 1:**
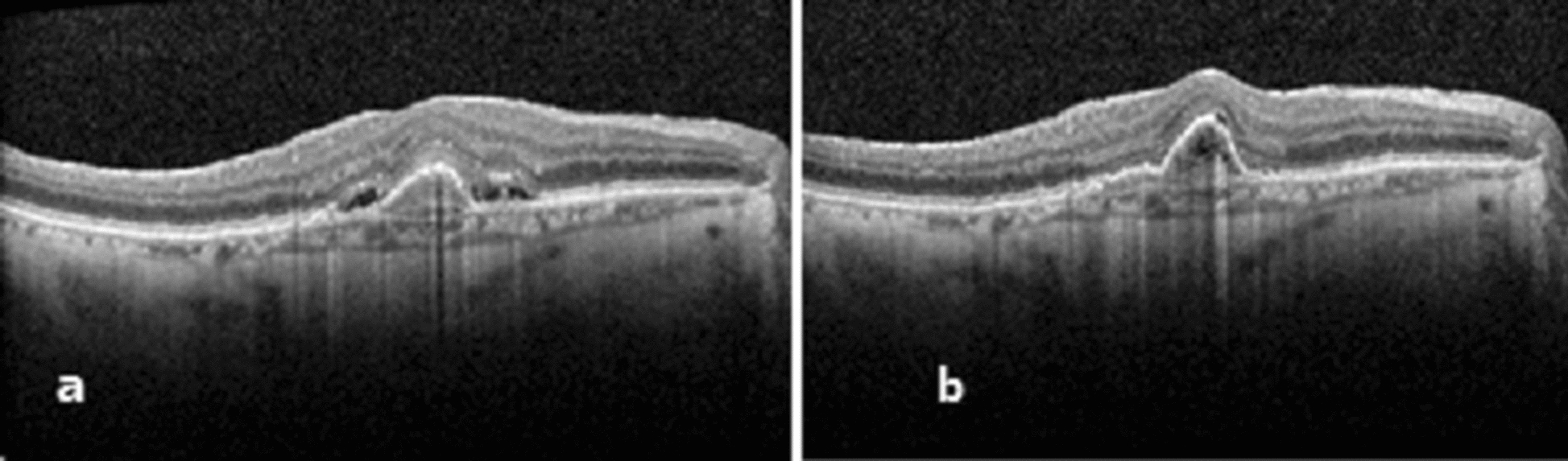
Response to treatment in patient without pachychoroid and pachyvessels. A 78 years old patient, who presented with metamorphopsia OD and o VA of 20\50 (0.4 LogMar). OCT showed a PED with SRF, thin choroid and no pachyvessels (**A**). OCT 4 weeks after three monthly bevacizumab injections showed complete resolution of the SRF but the PED enlarged a little, VA improved to 20\40 (0.3 LogMar) (**B**)

**Fig. 2:**
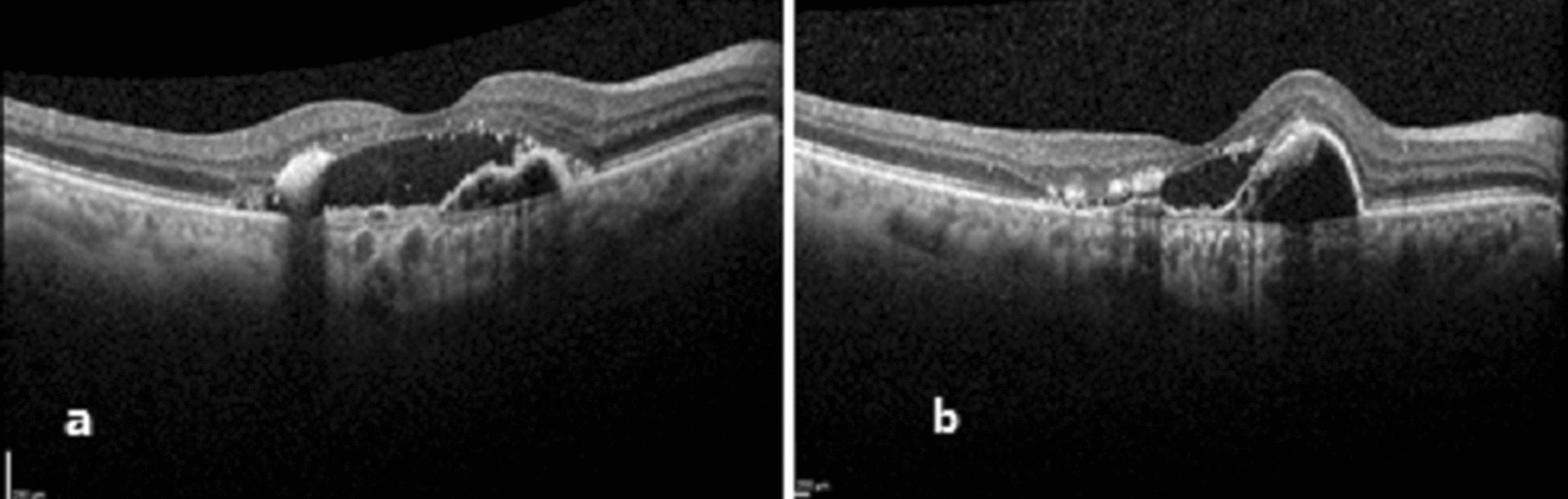
Response to treatment in patient with pachychoroid and pachyvessels. A 62 years old patient, who presented with a VA of 2.3 (LogMar), and OCT revealed pachychoroid of 370 μm with pachyvessels, a PED with SRF and hard exudates (**A**). OCT 4 weeks after three monthly bevacizumab injections showed improvement in the SRF, the PED enlarged. without change in the VA (**B**)

Response to treatment was compared between eyes with and without pachyvessels, as well as between eyes with and without increased choroidal thickness (analyzed with two cutoff values, above and below 250 and 300 μm). No correlation was found between the presence of pachyvessels or increased choroidal thickness and the improvement in VA, CMT or any of the other parameters (Table 2). Examples of patients with and without pachychoroid and pachyvessels are introduced in Figs. 1 and 2.

**Table 2:** Response to initial bevacizumab treatment regimen compared by choroidal thickness and presence of pachyvessels

	pachyvessels	Choroidal thickness above/bellow 250 µ	Choroidal thickness above/bellow 300 µ
Change in VA after 3 monthly injections (LogMar)	**No** − 0.24 ± 0.38 (P = 0.19)	** < 250 µ** − 0.13 ± 0.33(P = 0.77)	** < 300 µ** − 0.12 ± 0.30(P = 0.62)
	**Yes** − 0.081 ± 0.32(p = 0.19)	** > 250 µ** − 0.10 ± 0.34(P = 0.77)	** > 300 µ** − 0.06 ± 0.46(P = 0.62)
Change in CMT after 3 monthly injections (µ)	**No** − 119.3 ± 110.2 (P = 0.24)	** < 250 µ** − 91.63 ± 102.9(P = 0.57)	** < 300 µ** − 78.9 ± 103.4(P = 0.53)
	**Yes** − 75.7 ± 102.4 (P = 0.24)	** > 250 µ** − 73.85 ± 106.7(P = 0.57)	** > 300 µ** − 104.2 ± 110.07(P = 0.53)

The bold values are patient without pachyvessels

### Analysis of response to treatment at the final follow up period

Mean follow up duration was 13.3 ± 6.5 months, during which the mean number of injections was 21.5 ± 14.8. These included bevacizumab, aflibercept and ranibizumab that were administered following the first three initial bevacizumab injections. In 21 (46.6%) eyes, treatment was continued only with bevacizumab, 10 (22.2%) eyes were switched to aflibercept, 5 (11.1%) eyes were switched to ranibizumab and 9 (20%) eyes were treated with both aflibercept and ranibizumab after the three bevacizumab injections. Four (8.8%) eyes underwent cataract extraction during the follow up period. In 7 (15.5%) eyes foveal atrophy was noted at the final follow up.

At the final follow up, mean VA was 0.59 ± 0.6 logMAR, not significantly different than at presentation (p = 0.698). Central macular thickness was 305.2 ± 108.5 μm, remaining significantly reduced compared to the value at presentation (p < 0.001).

Rates of submacular hemorrhage remained significantly lower than at presentation (p = 0.001), but subretinal and intraretinal fluid were not. These results are presented in Table 1.

Again, there was no correlation between the presence of pachyvessels or increased choroidal thickness on the clinical or anatomical response to treatment. These analyses are presented in Table 3.

**Table 3:** Association between final outcomes to treatment (change in VA/CMT) and choroidal thickness/presence of pachyvessels

	pachyvessels	Choroidal thickness above/bellow 250 µ	Choroidal thickness above/bellow 300 µ
Change in VA between beginning and final follow up (LogMar)	**No** − 0.20 ± 0.77 (P = 0.097)	** < 250 µ** − 0.04 ± 0.65 (P = 0.44)	** < 300 µ** − 0.002 ± 0.50 (P = 0.68)
	**Yes** − 0.09 ± 0.39 (p = 0.097)	** > 250 µ** − 0.08 ± 0.37 (P = 0.44)	** > 300 µ** − 0.1 ± 0.5 (P = 0.68)
Change in CMT between beginning and final follow up (µ)	**No** − 126.36 ± 140 (P = 0.54)	** < 250 µ** − 81.9 ± 143.2(P = 0.29)	** < 300 µ** − 92.3 ± 133.5(P = 0.25)
	**Yes** − 96.18 ± 139.3 (P = 0.54)	** > 250 µ** − 126.9 ± 133.2(P = 0.29)	** > 300 µ** − 154.5 ± 158.4(P = 0.25)

The bold values are patient without pachyvessels

Eyes switched to aflibercept or ranibizumab injections showed better response in the central macular thickness in comparison to those in which bevacizumab injections were continued, with a mean change in CMT of 149.5 μm and 46.2 μm respectively (p = 0.013). There was no statistically significant difference between these groups regarding he change in the visual acuity and presence of IRF, SRF and hemorrhages.

No cases of endophthalmitis, vitreous hemorrhage or retinal detachment were encountered, and none of the patients required surgical intervention for complications of PCV or intravitreal injections.

## Discussion

PCV is likely an underestimated and under-diagnosed condition, especially in non-Asian patients. Several studies have compared the combination of anti-VEGF with PDT against the monotherapy of anti-VEGF. The results of the EVEREST-II stated that After 12 months, combination therapy of ranibizumab plus vPDT was superior to ranibizumab monotherapy in best-corrected visual acuity and superior in complete polyp regression while requiring fewer injections, in PLANET study patient treated with aflibercept monotherapy achieved visual and/or functional improvement in more than 85% of participants. As fewer than 15% met the criteria of a suboptimal response to receive PDT, the potential benefit of adding PDT could not be determined [18, 19].

However, studies comparing the different anti-VEGF agents are scarce, and it is not clear whether any anti-VEGF agent achieves better outcomes in these cases.

Gomi et al. [23] reported 3-month outcomes of intravitreal bevacizumab in 11 eyes with PCV. After treatments of one to three injections, most eyes demonstrated reduced exudative changes, but in only one eye were the polypoidal lesions fully resolved. Lai et al [24] also reported the efficacy of intravitreal bevacizumab therapy in 15 eyes with PCV. After the first 3 consecutive monthly 1.25 mg of intravitreal bevacizumab injections, significant improvement in both the vision and the foveal thickness was observed. Kumar et al. evaluated nine eyes of eight patients with naïve PCV who were treated with intravitreal bevacizumab monotherapy and showed that bevacizumab injection helps to improve or maintain the vision in patients with symptomatic treatment-naïve PCV [25].

The lack of head to head comparative studies of anti-VEGF monotherapy in PCV treatment makes the first regimen choice difficult. The only case series comparing two different types of anti VEGF monotherapy included 16 eyes with PCV and reported that the visual and anatomical outcomes in terms of PED reduction were better with aflibercept compared to bevacizumab. Patients treated with aflibercept improved in BCVA by approximately 1.5 lines at 6 months, whereas those treated with bevacizumab suffered a reduction in BCVA of approximately 2 lines [26].

Yamamoto et al*.* reported significant improvement of BCVA at 12 months with monotherapy of aflibercept [27]. Similarly, PLANET study results showed that aflibercept monotherapy was noninferior to combination of PDT + aflibercept, The gain of visual acuity was 10.7 versus 10.8 letters [19]. Previous reports have shown that switching over to aflibercept from ranibizumab may be helpful in resistant cases [28].

In our study all patients were treated with a fixed initial regimen of three monthly bevacizumab injections, and re-examined 4 weeks following the third injection. Visual acuity significantly improved with a mean gain of one line of vision after this initial treatment. Central macular thickness significantly improved too. The presence of subretinal fluid, intraretinal fluid and submacular hemorrhage also improved after the three injections. According to our results bevacizumab monotherapy was effective with good visual and anatomical outcomes after only three monthly injections, and could be reasonably used as a first line treatment in patients with PCV.

Additionally, we found that eyes that were later switched to aflibercept or ranibizumab injections showed better response in the central macular thickness in comparison to those in which bevacizumab injections were continued. There was no statistically significant difference between these groups regarding the change in the visual acuity and presence of IRF, SRF and hemorrhages. We note that it is possible that these cases were more resistant to treatment, however, patients in which response to bevacizumab treatment was inadequate were able to be switched to aflibercept or ranibizumab. These results do not indicate that these anti-VEGF agents are superior to bevacizumab, as their advantage was only in CMT reduction—a phenomenon well known in NVAMD as well as other conditions [25, 26].

Eyes with PCV have variable choroidal thickness, and prior studies showed conflicting results regarding the correlation between choroidal thickness, presence of pachyvessels and response to treatment [15–17] Some studies showed that pachychoroid have better clinical outcomes and some indicate the opposite. Our results show there was no correlation between the presence of pachyvessels or increased choroidal thickness on the clinical or anatomical response to treatment.

Limitations of this study include its retrospective nature and medium sample size. However, we note that this is a relatively large series of PVC in Caucasian patients, and emphasize the strength of their all having been treated uniformly upon diagnosis. Additionally, we note that PCV diagnosis was not based on FA and ICG in all cases. The final follow up period and the switching to second line treatment were variable and not fixed in all patients.

In conclusion, this is the largest study in non-Asian PCV patients treated with fixed first line treatment regimen with intravitreal bevacizumab monotherapy. Our results indicate that this regimen achieves satisfactory visual and anatomical outcomes. However, more resistant cases in which initial results were inadequate, showed better anatomical but not visual outcomes after switching to second line treatment with ranibizumab or aflibercept. Importantly, no correlation was found between choroidal thickness or presence of pachyvessels and response to treatment, making these findings better suited to be of diagnostic rather than prognostic value.

## Data Availability

The authors confirm that the data supporting the findings of this study are available within the article [and/or] its supplementary materials.

## Declarations

### Compteting interests

There is no Compteting interest for any of the authors.
